# Mogamulizumab as Bridge to Thiotepa‐Based Allogeneic Haematopoietic Stem Cell Transplantation in Sézary Syndrome: A Single‐Centre Experience

**DOI:** 10.1002/jha2.70331

**Published:** 2026-06-16

**Authors:** Simone Maifredi, Raffaella Sala, Chiara Bottelli, Chiara Pagani, Angela Passi, Rosa Daffini, Alessia Pantaleo, Domenico Russo, Michele Malagola, Marina Venturini, Daniele Avenoso, Alessandra Tucci

**Affiliations:** ^1^ Unit of Blood Diseases and Bone Marrow Transplantation Department of Clinical and Experimental Science University of Brescia ASST Spedali Civili di Brescia Brescia Italy; ^2^ Department of Dermatology University of Brescia ASST Spedali Civili di Brescia Brescia Italy; ^3^ Department of Haematology ASST Spedali Civili di Brescia Brescia Italy

**Keywords:** Allo‐HSCT, Mogamulizumab, Sézary syndrome

## Abstract

**Introduction:**

Sézary syndrome (SS) is an aggressive cutaneous T‐cell lymphoma for which allogeneic haematopoietic stem cell transplantation (allo‐HSCT) represents the only potentially curative treatment.

**Methods:**

We report a single‐centre series of three consecutive SS patients treated with mogamulizumab as bridging therapy to allo‐HSCT using thiotepa‐based reduced‐intensity conditioning.

**Results:**

Mogamulizumab induced rapid clearance of circulating Sézary cells and significant clinical improvement. All patients achieved early and stable full donor chimerism and complete remission. Post‐transplant GVHD occurred but was manageable with standard therapy.

**Conclusion:**

Early use of mogamulizumab may provide effective disease control and facilitate successful allo‐HSCT in SS.

**Trial Registration:**

The authors have confirmed clinical trial registration is not needed for this submission.

1

Sézary syndrome (SS) is a rare and aggressive leukemic variant of cutaneous T‐cell lymphoma (CTCL), characterized by generalized erythroderma with intense pruritus, lymphadenopathy and circulating malignant T cells with cerebriform nuclei. Reported 5‐year overall survival for Stage IV A1 cases is 48% [1], and allogeneic haematopoietic stem cell transplantation (allo‐HSCT) remains the only potentially curative option.

Mogamulizumab, a humanized monoclonal antibody targeting chemokine receptor type 4 (CCR4), has demonstrated efficacy in relapsed/refractory CTCL through antibody‐dependent cell‐mediated cytotoxicity against CCR4‐expressing malignant T cells. In the Phase 3 MAVORIC trial, which enrolled 372 patients with previously treated mycosis fungoides or SS, mogamulizumab achieved a significantly higher overall response rate (28% vs. 4.8%) and a longer median progression‐free survival (7.7 vs. 3.1 months) compared with Vorinostat [2]. On the other hand, by reducing the number of Tregs, where CCR4 is highly expressed, mogamulizumab can lead to a loss of immunotolerance, potentially increasing the risk of autoimmune reactions [3] or graft‐versus‐host disease (GVHD) after allo‐HSCT [4, 5].

For this reason, the optimal use of mogamulizumab as a bridging therapy to allo‐HSCT remains to be fully defined, particularly regarding timing and safety, in addition to disease control.

We report a single‐centre series of three consecutive female SS patients treated with mogamulizumab as a bridge to allo‐HSCT with thiotepa‐based reduced‐intensity conditioning. The classification system used was the 2018 WHO‐EORTC classification [6].

Mogamulizumab was administered according to the standard dose of 1.0 mg/kg intravenously once weekly during the initial 28‐day cycle, and subsequently on Days 1 and 15 of each following cycle. Conditioning regimens were reduced‐intensity and thiotepa‐based, in accordance with institutional protocols: TT‐FC [7] (thiotepa 10 mg/Kg, fludarabine 60 mg/m^2^, cyclophosphamide 60 mg/Kg) or miniTBF (thiotepa 5 mg/Kg D‐5; busulfan 3.2/mg/Kg Day D‐4,‐3,‐2; fludarabine 50 mg/m^2^ D‐4,‐3,‐2). Clinical characteristics are summarized in Table 1; longitudinal variations in immunophenotype and chimerism are presented in Figure 1.

**TABLE 1:** Clinical and therapeutic characteristics.

	**First case**	**Second case**	**Third case**
Sex	Female	Female	Female
Diagnosis	Sézary syndrome	Sézary syndrome	Sézary syndrome
Stage TNMB at diagnosis	IV A1	IV A1	IV A1
Age at diagnosis (years)	52	64	66
No. of previous therapies	II	I	II
No. of MOGA treatment cycles	14	6	3
Adverse effects before allo‐HSCT	No	No	No
Disease status at HSCT	PR	CR	PR
Time from diagnosis to HSCT (months)	30	13	15
Time from last MOGA to HSCT (days)	68	72	61
Donor	Sibling	MUD	Haplo‐HSCT
Conditioning	TT‐FC	TT‐FC	miniTBF
GVHD prophylaxis	ATG/CsA/MTX	PTCy/FK/MMF	PTCy/FK/MMF
aGVHD	Skin (Grade I)	Skin (Grade II)	No
cGVHD	No	Yes	No
Chimerism at +28 days from HSCT	Full	Full	Full
Flow cytometry at +28 days from HSCT	Neg	Neg	Neg
Last follow‐up from diagnosis (months)	57	27	22
Last follow‐up from HSCT (months)	27	14	7
Disease status at +100 days	CR	CR	CR
Disease status at last follow‐up	CR	CR	CR

*Note*: TT‐FC: thiotepa 10 mg/Kg, fludarabine 60 mg/m^2^, cyclophosphamide 60 mg/Kg; miniTBF: thiotepa 5 mg/Kg, busulfan 9.6 mg/Kg, fludarabine 150 mg/m^2^.

Abbreviations: aGVHD, acute graft‐versus‐host disease; ATG, anti‐thymocyte globulin; cGVHD, chronic graft‐versus‐host disease, CR, complete remission; CsA, cyclosporine A; FK, tacrolimus; HSCT, haematopoietic stem cell transplant; MMF, mycophenolate mofetil; MTX, methotrexate; MUD, matched unrelated donor; PR, partial remission; PTCy, post‐transplant cyclophosphamide.

**FIGURE 1:**
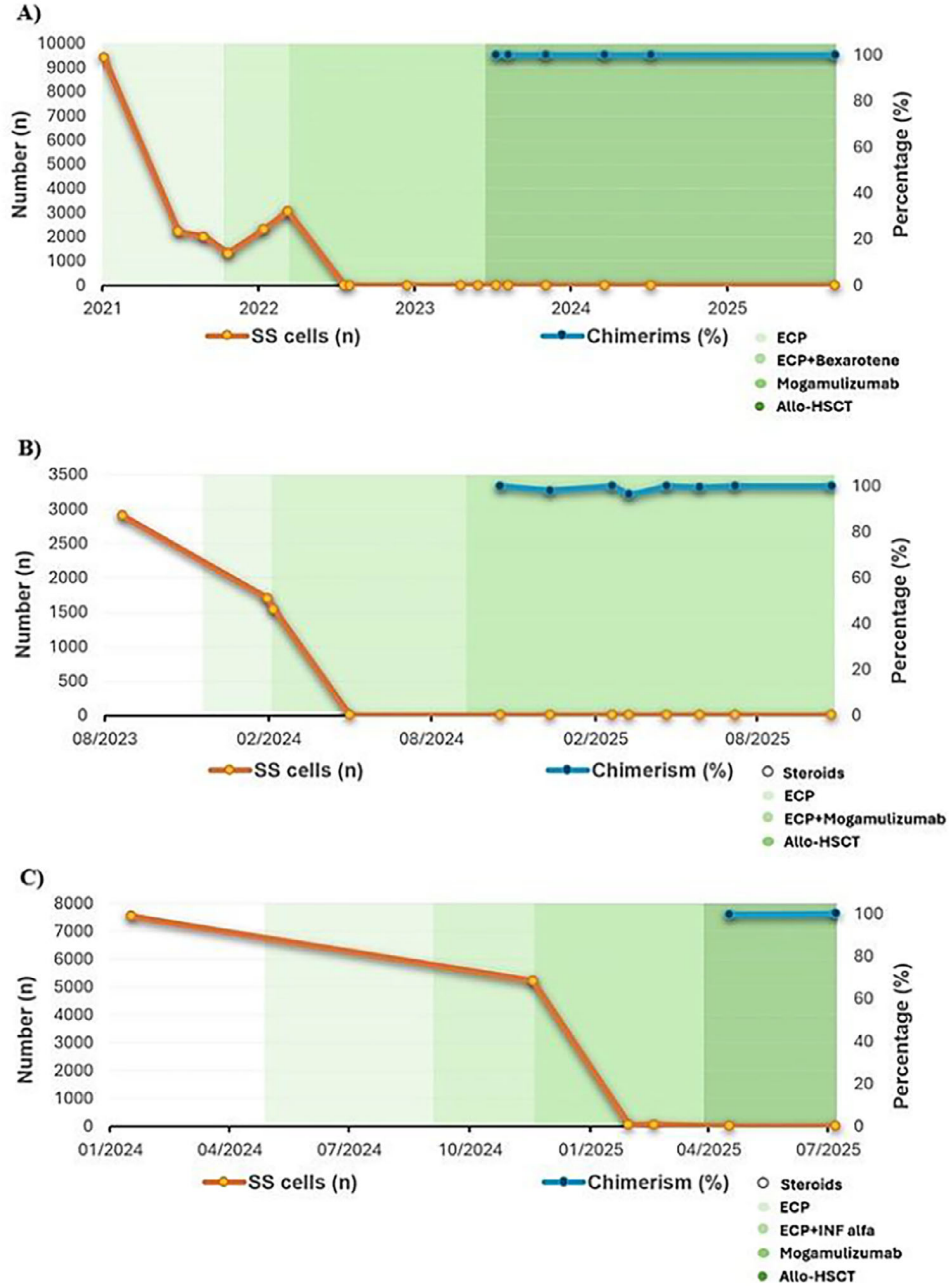
Immunophenotype and chimerism in the first (A), second (B) and third (C) patients.

The first patient, a 52‐year‐old woman, was referred for sub‐erythroderma and severe pruritus after a 7‐year history of erythematous patches initially treated as atopic dermatitis with PUVA (Psoralens+Ultraviolet A), corticosteroids, cyclosporine and methotrexate without benefit. Laboratory tests showed leucocytosis, elevated LDH and circulating convoluted lymphocytes; PET/CT revealed widespread hypermetabolic lymphadenopathies.

Flow cytometry (CD3^+^/CD4^+^/CD5^+^/CD45RO^+^/CD7^−^) and skin biopsy led to the diagnosis of SS, possibly evolved from previously undiagnosed mycosis fungoides, Stage IV A1 (T4NXM0B2). She was treated with extracorporeal photopheresis (ECP) and oral steroids, followed by bexarotene, without clinical improvement. Mogamulizumab induced rapid clearance of circulating Sézary cells and progressive skin improvement, but after 11 cycles, few erythematous pruritic lesions reappeared. Although clinical presentation and histology suggested mogamulizumab‐associated rash, T‐cell receptor clonality confirmed persistent disease. Given her good performance status and low tumour burden, allo‐HSCT with curative intent was proposed.

Sixty‐eight days after the last mogamulizumab infusion, the patient underwent TT‐FC conditioning followed by HLA‐matched allo‐HSCT; GVHD prophylaxis consisted of anti‐thymocyte globulin (Grafalon), cyclosporine and short‐course methotrexate. Early post‐transplant period was remarkable for suspected Stage I acute cutaneous GVHD that resolved with topical corticosteroids.

At the last follow‐up (+813 days), the patient is in complete remission, with full donor chimerism and no pathological cells detected by flow cytometry.

The second patient, a 64‐year‐old woman, was admitted for sub‐erythroderma with xerosis and plantar hyperkeratosis. Although the skin biopsy suggested a drug reaction, Stage IV A1 SS (T4N0M0B2) was confirmed by TCR clonality and flow cytometry of peripheral blood and bone marrow, showing a dominant CD4^+^ clonal population. She started ECP and, due to inadequate response after three cycles, mogamulizumab was added, resulting in rapid clinical remission and clearance of circulating malignant T cells. She underwent a matched unrelated donor (MUD) allo‐HSCT conditioned with TT‐FC after six cycles of mogamulizumab and a 72‐day washout. GVHD prophylaxis consisted of post‐transplant cyclophosphamide, tacrolimus and mycophenolate.

The first 100 days were remarkable for acute cutaneous GVHD (Grade II by MAGIC criteria) on Day +23, treated successfully with topical steroids and prednisone (1 mg/Kg/day). A flare on Day +108 required renewed steroids and subsequent ruxolitinib (10 mg/day), which achieved marked improvement, allowing rapid corticosteroid withdrawal. After 12 months, she developed moderate chronic GVHD (2014 NIH criteria: eyes and joints Grade 2; lungs Grade 1), treated with low‐dose prednisone (0.5 mg/Kg/day) with clinical benefit.

At the latest follow‐up (+420 days), the patient showed further GVHD improvement, full chimerism and no evidence of Sézary cells on immunophenotyping.

The third patient, a 66‐year‐old woman, was evaluated for diffuse erythroderma, severe pruritus and lymphocytosis. Peripheral blood smear revealed Sézary cells, and flow cytometry identified 56% of leukocytes expressing CD2/CD3/CD4/CD5/CCR7 and testing negative for CD8/CD26/CD7/CD45RA. Skin biopsy showed a mild CD3+ lymphocytic infiltrate with a slight predominance of CD4^+^ over CD8^+^ cells. CT/PET imaging showed multiple superficial hypermetabolic lymph nodes. Diagnosis was consistent with Stage IV A1 (T4NXM0B2) SS.

ECP was started with the addition of pegylated interferon‐α (90 µg/week) after 3 months due to only partial remission. Subsequently, 3 months later, due to disease progression, therapy was switched to mogamulizumab, with rapid skin improvement, reduced lymphadenopathy (< 2 cm) and a minimal residual blood clone (60 cells/µL).

After 3 cycles and a 61‐day washout, the patient underwent haploidentical HSCT following a mini‐TBF conditioning regimen; GVHD prophylaxis consisted of post‐transplant cyclophosphamide, tacrolimus and mycophenolate.

The early post‐transplant period was unremarkable. At the last follow‐up (Day +212), the patient is in complete remission with full donor chimerism and in good clinical condition.

These three cases represent the first reported consecutive mini‐series of patients who received mogamulizumab as a bridge to allo‐HSCT after a short history of SS and were managed with a completely chemo‐free approach. The optimal timing and the impact of prior therapies on transplant outcomes remain matters of debate. However, allo‐HSCT is still the only curative treatment for SS, even though high morbidity and mortality have been reported [8]. Previous experiences described patients who had received several lines of treatment, most of which included chemotherapeutic agents [9].In the Italian real‐life experience reported by Caruso et al. [9], as in our series, mogamulizumab demonstrated rapid and profound cytoreduction in the blood compartment, symptom control and feasibility as a bridge to allo‐HSCT. However, most patients reported by Caruso et al. underwent transplantation after multiple prior systemic therapies, and donor chimerism was often incomplete or delayed. Our series is characterized by an early integration of mogamulizumab and by the systematic use of thiotepa‐based reduced‐intensity conditioning. Notably, all patients in our cohort achieved early and stable full donor chimerism by Day +28. This finding suggests that the combination of pre‐transplant debulking with mogamulizumab and optimized thiotepa‐containing conditioning may synergistically promote full engraftment, a fundamental prerequisite for establishing an effective and durable graft‐versus‐lymphoma effect, which translates into long‐term disease control and potential cure. In our patients, post‐transplant complications were consistent with expected transplant‐related morbidity and were effectively controlled with standard interventions. The warning of increased GVHD risk after prior exposure to mogamulizumab has been especially reported in adult T‐cell leukaemia [10, 11] and appears to be mitigated by deferring transplantation for at least 50 days after the last dose.

These findings support the feasibility and benefit of early integration of mogamulizumab into pre‐transplant treatment algorithms for SS, in combination with optimized conditioning strategies. The results of an ongoing observational study (NCT04014374) may clarify optimal timing, long‐term safety and the impact of mogamulizumab on immune reconstitution and GVHD risk after allo‐HSCT.

## Funding

The authors have nothing to report.

## Conflicts of Interest

The authors declare no conflicts of interest.

## Data Availability

The data that support the findings of this study are available on request from the corresponding author. The data are not publicly available due to privacy or ethical restrictions.
